# Blood Essential Trace Elements and Alzheimer's Disease Biomarkers in Midlife

**DOI:** 10.1002/alz70860_099781

**Published:** 2025-12-23

**Authors:** Xin Wang, Kelly M. Bakulski, Carrie A. Karvonen‐Gutierrez, Sung Kyun Park, David Morgan, Brian P Jackson, Roger L. Albin, Henry L Paulson

**Affiliations:** ^1^ University of Michigan, Ann Arbor, MI, USA; ^2^ Michigan State University, Grand Rapids, MI, USA; ^3^ Dartmouth College, Hanover, NH, USA

## Abstract

**Background:**

Alzheimer's Disease (AD) is a progressive neurodegenerative disorder characterized by cognitive decline, impacting millions globally. Essential trace elements are implicated in key age‐related physiologic processes but have not been fully examined with respect to AD etiology. This study investigates associations between serum levels of essential trace elements (manganese, iron, cobalt, copper, zinc, selenium, and molybdenum) and AD biomarkers (Aβ42, Aβ42/Aβ40 ratio, *p*‐tau181, and total tau) in midlife women.

**Method:**

This cross‐sectional study included 194 midlife women (median age=53.3 years) from the Study of Women's Health Across the Nation, Michigan site. Serum levels of trace elements were measured using inductively coupled plasma‐mass spectrometry, and AD biomarkers were quantified using single molecule array assays. Multivariable linear regression models assessed potential associations and Bayesian kernel machine regression (BKMR) was used to account for complex co‐exposures and non‐linear relationships.

**Result:**

In the multivariable linear regression models, a doubling of serum molybdenum level was associated with 9.4% higher Aβ42/40 ratio (95% CI: 0.8%, 18.6%; *p* = 0.03), and a doubling of serum cobalt level with 17.5% higher *p*‐tau181 level (95% CI: 3.1%, 33.8%; *p* = 0.02). Copper showed an inverse association with the Aβ42/40 ratio, while zinc was positively associated with the Aβ42/40 ratio, though these associations were marginally significant. BKMR analysis confirmed these associations.

**Conclusion:**

This study identified statistically significant associations of serum molybdenum and cobalt levels with AD biomarkers, suggesting a potential protective effect of molybdenum against Aβ aggregation and exacerbation of pathologic tau phosphorylation by cobalt. These findings underscore the need for further longitudinal studies to explore the role of essential trace elements in AD pathogenesis.

